# Enhanced Recovery of Phenolic and Tocopherolic Compounds from Walnut (*Juglans Regia* L.) Male Flowers Based on Process Optimization of Ultrasonic Assisted-Extraction: Phytochemical Profile and Biological Activities

**DOI:** 10.3390/antiox10040607

**Published:** 2021-04-15

**Authors:** Anca Pop, Ionel Fizeșan, Laurian Vlase, Marius Emil Rusu, Julien Cherfan, Mihai Babota, Ana-Maria Gheldiu, Ioan Tomuta, Daniela-Saveta Popa

**Affiliations:** 1 Department of Toxicology, Faculty of Pharmacy, Iuliu Hatieganu University of Medicine and Pharmacy, 8 Victor Babes, 400012 Cluj-Napoca, Romania; anca.pop@umfcluj.ro (A.P.); ionel.fizesan@umfcluj.ro (I.F.); dpopa@umfcluj.ro (D.-S.P.); 2 Department of Pharmaceutical Technology and Biopharmaceutics, Faculty of Pharmacy, Iuliu Hatieganu University of Medicine and Pharmacy, 8 Victor Babes, 400012 Cluj-Napoca, Romania; tomutaioan@umfcluj.ro; 3 BCBS Team (Biotechnologies et Chimie des Bioressources Pour la Santé), LIENSs Laboratory (Littoral Environment et Sociétés), UMR CNRS 7266, University of la Rochelle, 17000 La Rochelle, France; julien.cherfan@univ-lr.fr; 4 Department of Pharmaceutical Botany, Faculty of Pharmacy, Iuliu Hatieganu University of Medicine and Pharmacy, 8 Victor Babes, 400012 Cluj-Napoca, Romania; mihai.babota@umfcluj.ro (M.B.); gheldiu.ana@umfcluj.ro (A.-M.G.)

**Keywords:** walnut male flowers, polyphenols, tocopherols, antioxidants, LC-MS, biological activity, experimental design, enzymatic inhibition, anticancerous, ultrasound-assisted extraction

## Abstract

The extraction of bioactive compounds present in walnut (*Juglans regia* L.) male flowers (WMFs) was performed based on an experimental design using ultrasonic-assisted extraction. Solvent nature, extraction time, and water content were selected as experimental variables, and phenolic, flavonoidic, and condensed tannins contents and antioxidant properties were evaluated. Acetone was the solvent with the highest extraction performance, with the extracts obtained using this solvent displaying an increased concentration of bioactive compounds and increased antioxidant activities. For several extracts with high bioactive content, individual polyphenolic and tocopherolic compounds were evaluated by means of LC-MS and LC-MS/MS. The best extraction conditions for polyphenolic (2.86 mg gallic acid equivalents/g WMF) and tocopherolic compounds (29.4 µg/g WMF) were acetone with 40% water content (N20) and acetone with 20% water content (N15), respectively. Although the total tocopherol concentrations were lower than in other *Juglans regia* parts, most of the total tocopherol quantity was provided by the highly biologically active δ-tocopherol (84%). Significant quantities of quercetin (101.9 µg/g), hyperoside (2662.9 µg/g), quercitrin (405.7 µg/g), and isoquercitrin (1293.7 µg/g) were determined in WMF (N20). Both extracts inhibited the enzymatic activity of α-glucosidase and tyrosinase; however, an increased inhibition was observed for N20, the extract with the higher polyphenolic content. Conversely, N15 had higher anticancerous activity on the cell lines used, with a moderate selectivity towards the cancerous phenotype being observed for both extracts. At non-cytotoxic concentrations, both extracts displayed good antioxidant activities in cellular cultures, decreasing basal and H_2_O_2_-induced oxidative stress. This is the first characterization of both hydrophilic and lipophilic phytochemicals in WMF extracts. The outcomes of our study reveal that walnut male flowers have strong biological activities, thus justifying further research to demonstrate their usefulness in the food, pharmaceutical, and/or cosmetic industries.

## 1. Introduction

The English or common walnut (*Juglans regia* L.), belonging to Juglandaceae family, is an important source of nutrients and bioactive compounds, especially phenolics, with notable antioxidant activity and therapeutic potential. The walnut tree has a long history of medicinal use, being widely used in traditional medicine in many countries [1,2]. Walnuts, due to their content of protein, fibers, essential fatty acids, vitamins and minerals, as well as other phytochemicals, are ingredients in different diets recommended to prevent aging and age-related diseases [3], and also in the nutrition of people with cardiovascular or neurodegenerative conditions, type II diabetes or cancer [1,4,5]. Other parts of *J. regia* are natural sources of bioactive compounds utilized in phytotherapy, the food and pharmaceutical industries, and in cosmetic products [6]. The leaf and green husk extracts have demonstrated antioxidant and anti-inflammatory activities; antidiabetic effects; and astringent, antiseptic, and anthelmintic properties [2,7]. The walnut septum contains bioactive compounds with antioxidant, anti-inflammatory, antitussive and antidiabetics properties and has a high potential for hematologic regeneration and anti-aging activity, demonstrated both in vitro [8,9,10] and in vivo [10,11,12,13,14].

Walnut male flowers (WMFs) are included in the traditional diets of some Chinese regions, where they are known as longevity foods [15,16]. In traditional medicine, WMFs are used in malaria and rheumatic pain treatments [2]. In recent years, several studies have focused on the investigation of the phytochemical profile of WMFs and their biological activities. Rich in carbohydrates, protein, lipids and minerals (potassium, manganese, copper, iron, zinc, calcium, magnesium) [15,17], WMFs represent an interesting vegetal matrix for the development of new food products and bioactive molecule extraction. In one of the first studies on WMFs, a methanolic extract displayed antioxidant, antihypoxic, anti-inflammatory and antidepressive activities in a rodent model [18]. Ebrahimzadeh et al. reported antihemolytic activity of the same extract, whereas Hosseini et al. revealed antidiabetic properties of a hydroalcoholic extract in streptozotocine-induced diabetes in mice [17,19]. Recently, Muzzafer et al. reported that WMF extracts present antioxidant, antibacterial and photoprotective effects on human skin cells exposed to UVB light [20].

Considering these findings, there is an interest in the isolation/identification and quantification of active constituents from WMFs to investigate and explain their pharmacological activities [1]. There are different extraction methods reported in the literature, applied and tested to obtain WMF extracts that are rich in nutrients and/or phytochemicals. Among the solvents (methanol, ethanol and water) employed to prepare cold macerated extracts, the methanolic extract was found to be the richest in phytochemicals (phytosterols, alkaloids, saponins, phenolics and flavonoids) [20].

Due to the limited information on the phytochemical profile and biological activities of WMF, we intend to extend the existing scientific data about this promising by-product. The main aims of our study were (1) to investigate the efficiency of different solvents and experimental conditions on the Romanian walnut flower matrix, using the design of experiments (DoE) technique; (2) to obtain extracts rich in bioactive compounds with strong antioxidant activity; (3) to characterize the phytochemical profiles of the optimum extracts by means of liquid chromatography–mass spectrometry (LC-MS) and liquid chromatography coupled with tandem mass spectrometry (LC-MS/MS) methods; (4) to assess the biological activity of the optimum extract in terms of its inhibitory activity on a selection of enzymes and its cytotoxic and antioxidant effects on different cancer and normal cell lines.

## 2. Materials and Methods

### 2.1. Chemicals

All reagents and standards used were of analytical grade. The reagents used in this study were diammonium 2,2′-azino- bis(3-ethylbenzothiazoline-6-sulfonate) (ABTS) (>98%), 3,4-dihydroxy-L-phenylalanine (L-DOPA) (≥98%), dimethyl sulfoxide (DMSO) (≥99%), 2,2-diphenyl-1-(2,4,6-trinitro-phenyl) hydrazine (DPPH), ferric chloride, α-glucosidase solution (Saccharomyces cerevisiae, EC 3.2.1.20), 6-hydroxy-2,5,7,8-tetramethylchromane-2-carboxylic acid (Trolox) (97%), kojic acid, mushroom tyrosinase, phosphate buffer, sodium carbonate, 2,4,6-tris(2-pyridyl)-S-triazine (TPTZ) (≥99%) and vanillin (99%), which were purchased from Sigma (Sigma Aldrich Chemie GmbH, Schnelldorf, Germany).

All the standards used for both LC-MS and LC-MS/MS analyses were purchased from Sigma-Aldrich (Schnelldorf, Germany) except for ferulic acid (≥99%) and gallic acid (≥98%), which were purchased from Merck (Darmstadt, Germany), and δ-tocopherol from Supelco (Bellefonte, PA, USA). Aluminum chloride (≥98%) was provided by Carl Roth (Karlsruhe, Germany), and Folin–Ciocâlteu reagent; the solvents acetone, ethanol and methanol; as well as hydrochloric acid (37%) were supplied by Merck (Darmstadt, Germany). Water was of Milli-Q-quality.

### 2.2. Preparation of Walnut Flower Extract

High-grade WMFs were obtained from a local orchard in Cluj County, Romania, in early May 2019. The sample trees, around 10 years old, are located in north-western Romania (46°46′ N, 23°35′ E, 360 m altitude). The plant material was identified by Dr. Ana-Maria Gheldiu from the Department of Pharmaceutical Botany, Iuliu Hatieganu University of Medicine and Pharmacy Cluj-Napoca, Romania, and a voucher specimen was kept in the herbarium of this department. The WMFs were dried in the shade for 3 days at room temperature (RT) and stored in the refrigerator until extraction.

#### Preparation of the Extracts

The flowers were ground in a coffee grinder for 5 min and then passed through a 200-µm Retsch sieve. WMF powder was measured (1 g) and mixed with the solvent (10 mL) in glass tubes with stoppers. Ultrasonication (US) was accomplished using an ultrasonic bath (Elma Transsonic 700/H, Singen, Germany) for 10, 30 and 50 min. The homogenates were centrifuged (Hettich, Micro 22R, Andreas Hettich GmbH and Co., Tuttlingen, Germany) for 10 min at 5000 rpm and the resulting supernatant was separated. Consecutively, the solvent of the crude extract was separated under vacuum at 45 °C using a rotary evaporator (Hei-VAP, Heidolph Instruments GmbH and Co., Schwabach, Germany) and the resulting residue was taken up in water and lyophilized (Advantage 2.0, SP Scientific, Warminster, PA, USA). If not stated otherwise, the lyophilized extracts dissolved in 70% EtOH (10 mg/mL) were used to perform the analyses.

### 2.3. Experimental Design

The role of several parameters on the extraction efficiency, together with their possible interactions, was investigated based on the DoE technique. Optimization of the extraction procedure was accomplished by applying the response surface methodology (RSM), a recognized and widely used collection of statistical tools. DoE offers the largest amount of data from the least number of experiments by systematic variation of multiple factors and the coordinated evaluation of the resulting effects. The DoE technique consists of several steps: description of the experimental objective, designation of the factors and responses significant to the goals of the experiment, selection of the regression model and generation of the experimental design [21].

A D-optimal DoE, selected for the accurate assessment of factor effects and the minimum number of experimental runs, can be used in several situations, such as in the investigation of multi-level qualitative factors or when an optimal number of runs is desired. For regression analysis, the following parameters were considered: R^2^, Q^2^, model validity and reproducibility.

R^2^ represents the goodness of fit of a model and represents the response variance explained by the model. It is computed based on the equation:

R^2^ = 1 − SSres/SStot corr

where SSres is the amount of variation that is not modeled and SStot corr is the total corrected variation in response.

Q^2^ signifies the capacity of prediction and reveals how well the selected model can predict new experiments. Q^2^ is calculated according to the equation:

Q^2^ = 1 − SSpres/SStot corr

where SSpress is the prediction error sum of squares and SStot corr is the total corrected variation in response [22].

All the steps and calculations of the D-optimal design were completed with Modde version 11.0 (Sartorius Stedim Data Analytics AB, Umeå, Sweden) software. Three factors—extraction time, solvent and water content (%) in the solvent—were the independent variables for the D-optimal experimental design. The total phenolic content (TPC), total flavonoid content (TFC), condensed tannin content (CTC), 2,2-diphenyl-1-picrylhydrazyl (DPPH), ferric reducing antioxidant power (FRAP) and trolox equivalent antioxidant capacity (TEAC) assays were the dependent variables (Table 1).

### 2.4. Determination of Total Bioactive Compounds

#### 2.4.1. Total Phenolic Content

The TPC of the WMF crude extracts was measured using the Folin-Ciocâlteu (FC) spectrophotometric method, according to a previously described method [23].

Each sample (20 µL) was incubated with FC reagent (100 µL) for 3 min and subsequently with of 7.5% sodium carbonate (80 µL). The resulting solutions were incubated in the dark at RT for 30 min and the absorbance was measured at 760 nm against a solvent blank. The reference standard was gallic acid and the TPC was calculated as gallic acid equivalents (GAE) per gram of dry weight (dw) plant.

#### 2.4.2. Total Flavonoid Content

The TFC of the WMF crude extracts was measured according to a previously described method [24]. The crude extract (100 µL) was mixed with 2% aluminium chloride (100 µL) and further incubated in the dark at RT for 15 min. The absorbance was registered at 420 nm against a solvent blank and the TFC was calculated as quercetin equivalents (QE) per gram of dw plant.

#### 2.4.3. Condensed Tannin Content

The CTC in WMF crude extracts was measured according to a previously described vanillin assay [25]. In a 96-well plate format, a volume of 50 µL of each sample was incubated with 250 µL of a solution containing 0.5% vanillin dissolved in 4% HCl in methanol. The plate was kept for 20 min in the dark at 30 °C and the absorbance was assayed at 500 nm. The CTC was calculated as catechin equivalents (CE) per gram of dw plant.

### 2.5. Determination of the Antioxidant Activity

#### 2.5.1. DPPH Radical Scavenging Activity

The antiradical activity of the WMF crude extracts against the free radical DPPH was calculated as previously described [26]. Each sample (30 µL) was incubated for 30 min in the dark with DPPH (0.004% methanol solution). The absorbance was subsequently measured at 517 nm against a solvent blank and the obtained results were conveyed as Trolox equivalents (TE) per gram of dw plant.

#### 2.5.2. FRAP Assay

The antioxidant capacity of the WMF crude extracts was measured by means of a FRAP assay that evaluates the reduction of Fe^3+^-TPTZ to Fe^2+^-TPTZ, a blue-colored compound. A minor modified described method was applied [27]. Briefly, each sample (25 µL) was added to FRAP reagent (175 µL) and further incubated for 30 min in the dark. The absorbance was assessed at 593 nm and the data were calculated as Trolox equivalents (TE) per gram of dw plant.

#### 2.5.3. TEAC Assay

The antiradical activities of the WMF crude extracts were assessed based on a TEAC test, as mentioned earlier [28]. Each sample (20 µL) was mixed with a 2,2′-azino-bis(3-ethylbenzothiazoline)-6-sulphonic acid (ABTS) radical solution (200 µL), and the absorbance was measured at 760 nm after 6 min of incubation. The outcomes were expressed as Trolox equivalents (TE) per gram of dw plant.

### 2.6. Analyses of the WMF Extracts

Based on the experimental results obtained during the previous steps, the six extracts with the highest concentration of polyphenols and the most prominent antioxidant activity were selected to be further analyzed for individual phenolic compounds and tocopherol content. All determinations were performed in triplicate (n = 3).

#### 2.6.1. Individual Phenolic Investigation

An LC-MS method, previously described, was used to analyze the phenolic composition of the WMF crude extracts [29]. Briefly, an Agilent 1100 HPLC Series system (Agilent, Santa Clara, CA, USA) and a UV detector coupled with an Agilent Ion Trap 1100 SL mass spectrometer (LC/MS Ion Trap VL) were used, and the chromatographic separation was done on a Zorbax SB-C18 column (100 mm × 3.0 mm i.d., 3.5 µm) (Agilent Technologies, Santa Clara, CA, USA) with a solvent mixture containing methanol/acetic acid 0.1% (*v*/*v*). A linear gradient from 5% to 42% methanol for a period of 35 min was followed by an isocratic elution for 3 min and a rebalancing with 5% methanol for 7 min. Five microliters of samples were injected and the separation was performed with a solvent flow rate of 1 mL/min at 48 °C. The detection process was accomplished in UV and MS mode. The detection of polyphenolic acids was performed during the initial 17 min of the chromatographic separation and the wavelength of the UV detector was set at 330 nm, whereas the detection of flavonoids and their aglycones was performed at 370 nm until the end of the separation. For the MS detection, the system operated with the electrospray ion (ESI) source in negative mode (capillary 3000 V, nebulizer 60 psi (nitrogen), dry gas temperature 360 °C, and dry nitrogen gas at 12 L/min). A previously-described complementary LC-MS method was employed to analyze another set of polyphenols: gallic acid, syringic acid, vanillic acid, protocatechuic acid, catechin and epicatechin [10]. The chromatographic separation was performed under the same conditions but with a slightly different binary gradient (0–3 min: 3% methanol; 3–8.5 min: 8% methanol; 8.5–10 min: 20% methanol; at 10 min: rebalance column with 3% methanol).

All compounds were assessed based on their peak areas and standard calibration curves. The data were calculated as micrograms of phenolic compounds per gram of dw plant (µg/g).

#### 2.6.2. Tocopherol Quantification

The tocopherol content was investigated using a previously validated LC-MS/MS method [10]. The same HPLC system, coupled with a Brucker Ion Trap SL (Brucker Daltonics GmbH, Leipzig, Germany) and a chromatographic column were used. The separation was performed using a mobile phase from water/methanol (7:93; *v*/*v*), isocratic elution and an injection volume of 10 µL (for both standard solution and crude extract) at 40 °C, with a flow rate of 1 mL/min. The MS detection was done using an atmospheric pressure chemical ionization (APCI) source and negative ionization in multiple-reaction monitoring (MRM) mode. The standard solutions (1 mg/mL) of the tocopherols were prepared in methanol and the working solutions were prepared by dilutions in water/acetone (50:50, *v*/*v*). Calibration curves with correlation coefficients r greater than 0.99 and linearity from 40 to 960 ng/mL were obtained for all evaluated tocopherols.

### 2.7. Biological Activities

Based on the phytochemical profiling (individual polyphenols and tocopherols) and the in vitro abiotic antioxidant assays, two extracts with increased performances were selected to further study their biological activities. The extract N20, obtained using acetone with 40% water content, and extract N15, obtained using acetone with 20% water content, were evaluated for their biological activities, including their enzyme inhibitory potential and their anticancerous and antioxidant activities on cell cultures.

#### 2.7.1. Enzyme Inhibitory Assays

##### Tyrosinase Inhibitory Assay

The inhibitory activity of the WMF crude extracts on tyrosinase was assayed as previously described [30]. The WMF extracts were dissolved in water containing 5% DMSO. In a 96-well plate format, four wells were designated as follows: (A) 46 U/mL (40 µL) mushroom tyrosinase (MT) in 66 mM phosphate buffer (PB), pH 6.6 (120 µL); (B) only PB (160 µL); (C) PB (80 µL), MT (40 µL) and the sample (40 µL); (D) PB (120 µL) and the sample (40 µL) incubated at RT for 10 min. Then, 2.5 mM L-DOPA prepared in PB (40 µL) was added into each well and incubated at RT for 20 min. The absorbance of the colored solution was measured spectrophotometrically at 475 nm. Kojic acid was used as an external standard to calculate the enzymatic inhibitory activity of the extracts. The inhibition was calculated based on the following formula ((A − B) − (C − D)) × 100/(A − B) and the results were calculated as mg kojic acid equivalents (KAE)/g of dw plant.

##### α-Glucosidase Inhibitory Assay

The inhibitory activity of the WMF crude extracts on α-glucosidase was assayed as previously described [31]. Briefly, each sample (50 µL) was mixed with glutathione (0.5 mg/mL) (50 µL), 10 mM PNPG (*p*-nitrophenyl-β-d-glucuronide) (50 µL) and α-glucosidase in PB (50 µL) and incubated for 15 min at 37 °C. The enzymatic reaction was stopped after the incubation period by adding 0.2 M sodium carbonate (50 µL) and the absorbance was measured at 400 nm. The results were expressed as mg acarbose equivalents (ACAE)/g of dw plant.

#### 2.7.2. Biological Activities of WMF Extract on Cell Lines

##### Cell Culture

The normal human foreskin fibroblasts (BJ) and the cancerous derived human cell lines A549 (lung adenocarcinoma), T47D-KBluc and MCF-7 (breast cancers) (ATCC, Manassas, United States of America) were used to assess a possible anticancerous effect. BJ and A549 cells were cultivated in DMEM (Dulbecco’s modified Eagle’s medium) supplemented with 10% FBS (fetal bovine serum), whereas MCF-7 and T47D-KBluc cells were cultivated in RPMI 1640 (Roswell Park Memorial Institute Medium) supplemented with 10% FBS. Cells were maintained at 37 °C in a humidified incubator with a 5% CO_2_ atmosphere. Medium was replaced every two days and the cells were used in experiments or subcultured at around 70%–80% confluency.

##### Preparation of Extract Solutions

Working solutions with N20 and N15 lyophilized extracts were prepared in DMSO at concentrations ranging from 0.25 to 100 mg/mL. The selected extracts were evaluated on cell cultures at concentrations ranging from 20 to 800 µg/mL by diluting the DMSO solutions with the appropriate cell culture media.

##### Viability Assays

Trypsinized cells were seeded in 96-well plates and left to attach for 24 h. After 24 h, cell debris was washed with phosphate-buffered saline (PBS), while the viable cells were incubated for 24 h/48 h with the two extracts at different concentrations. Subsequently, the cells were washed with PBS and viability was assessed by means of the Alamar Blue assay. The fluorescence intensity was measured at λ_excitation_ = 530/25, λ_emission_ = 590/35 after an incubation period of 3 h with a 200-µM resazurin solution, using a Synergy 2 multi-mode microplate reader [32]. The viability was expressed as relative viability compared to the negative control (100%) (0.2% DMSO). To facilitate comparisons between the conditions tested, 50% inhibitory concentration (IC_50_) values were derived from the dose-response curves obtained using a four-parameter logistic curve.

##### Dichloro-Fluorescein Diacetate (DCFH-DA) Assay

The antioxidant capacity of the two extracts was evaluated on the normal cells using 2,7 dichloro-fluorescein diacetate (DCFH-DA) dye. The cells were incubated for 24 h to three non-cytotoxic concentrations of N20 and N15 and N-acetyl cysteine (NAC), used as an antioxidant reference compound. The cells were further washed with PBS to remove any traces of serum and were incubated for 2 h with 50 µM DCFH-DA dye dissolved in Hanks’ balanced salt solution (HBSS). Following the incubation, another wash with PBS was performed and cells were exposed to 250 µM H_2_O_2_ for 3 h. The fluorescence intensity was evaluated using a Synergy 2 multi-mode microplate reader at λ_excitation_ = 485/20, λ_emission_ = 528/20 [32].

### 2.8. Statistical Analysis

The data are expressed as mean values ± standard deviation (SD) and, unless stated otherwise, the normally distributed result sets were evaluated using one-way analysis of variance (ANOVA). Data analyses and graphical representation were performed in SigmaPlot 11.0 computer software (Systat). Results with *p*-values ˂ 0.05 were considered statistically significant.

## 3. Results and Discussion

### 3.1. Fitting the Experimental Data

The 21 formulations generated by the software and the outcomes obtained after performing all experiments are specified in Table 2.

**Figure 1:**
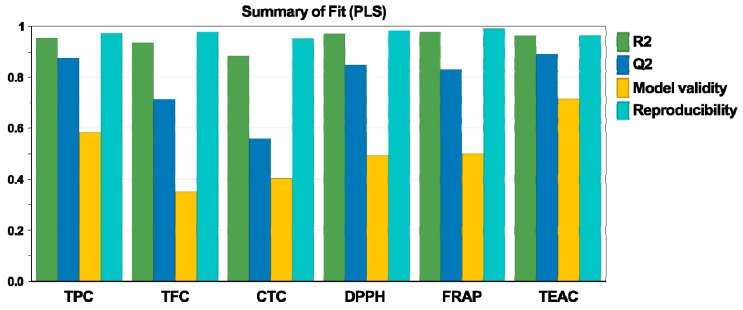
Fitting of experimental data using the partial least squares (PLS) model.

The experimental design was fitted employing the partial least squares (PLS) model. According to Figure 1, the values of R^2^ and Q^2^ were greater than 0.5, demonstrating high goodness of fit and power of prediction [33]. Moreover, the reproducibility values and the model validity were greater than 0.9 and 0.35, respectively, suggesting that the experimental setup was correct and that by working under the same conditions, comparable responses would be obtained.

The analysis of variance (ANOVA) test is another important diagnostic test for model validity (Table 3). It displays whether the response variance is determined by factor modifications or by experimental errors [34]. For all dependent variables, the *p*-values of the ANOVA test were ˂0.05 for the model and ˃0.05 for the lack of fit. These outcomes demonstrate that the quadratic model is statistically good and indicate a significant influence of the factors over the responses.

The regression coefficients were ˃ 0.90 for most of the responses; thus, a high percentage of the response variance was explained by the model. At an R^2^ value of 0.90, 90% of the response was explained by the model [35]. The Q^2^ values ˃ 0.5 and the small differences ˂0.2 between R^2^ and Q^2^, achieved for five responses, show good predictive power. Considering the results, the chosen model was found to be appropriate to describe the experimental data, as the values for the lack of fit were not significant in relation to the pure error.

### 3.2. The Influence of Experimental Conditions

The interpretation of the model can be easily performed through regression equation coefficients (Table 4).

#### 3.2.1. The Influence of Experimental Conditions on TPC, TFC and CTC

Various studies have demonstrated the rich phenolic content of different types of nuts and the relationship between the phenolic profile, antioxidant activity and the health benefits they provide in human populations of all ages [36]. Tree nut by-products compared to kernels provide most of the polyphenols, hence the scientific focus on this research field [37,38]. Our team previously revealed the potential of some other by-products [8,10,39].

In this experiment, we concentrated on a less studied plant matrix. The outcomes (Table 2) demonstrate the influence of the factors used in the experimental design. The extraction intervals tested were 10, 30 and 50 min and the three solvents—methanol, ethanol and acetone—were mixed with water in various proportions (20%, 40% and 60%). One of the most commonly selected independent variables in vegetable extraction techniques is the solvent. We decided on three polar solvents, which are regarded as the best option for better results in phenolic extraction. As expected, based on previous studies [8], the mixture of acetone and water—a polar aprotic relatively acidic solvent and a polar protic solvent, respectively—can be more efficient than a mixture of two polar protic solvents, water and ethanol or methanol.

**Figure 2:**
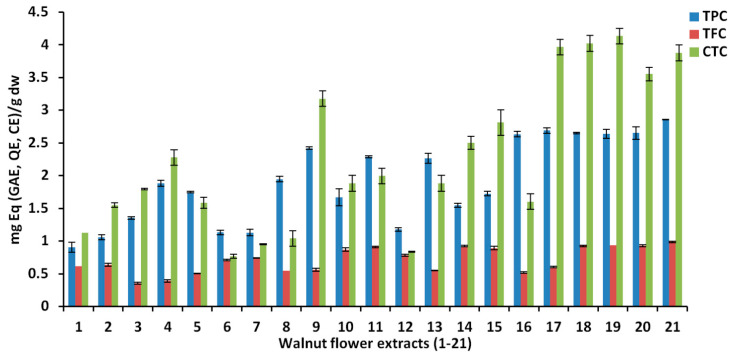
Total phenolic content (TPC), total flavonoid content (TFC) and condensed tannin content (CTC) of analyzed walnut flower extracts.

The TPC, TFC and CTC results for the 21 WMF extracts are presented in Figure 2. The best outcomes were detected for 30 min extraction time and 40% water in acetone. For TPC, the highest amount was 2.86 mg GAE/g (Table 2), which was around the content found by Rosa et al. [40], but less than the value of 24.3 mg GAE/g obtained from the same matrix in another study [15]. There could be many reasons for these discrepancies, as already mentioned, such as the walnut cultivar, the age of the tree, the different flowering stages or the geographic location. The same is applicable for the TFC and CTC amounts.

**Figure 3:**
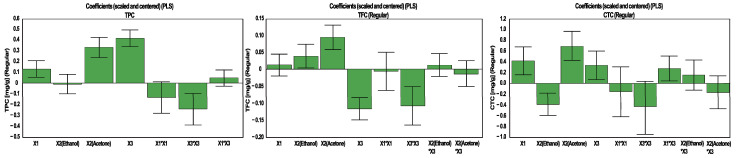
Influence of working conditions on the TPC, TFC and CTC in walnut flower extracts, presented as scaled and centered coefficient plots. X_1_—extraction time (min); X_2_—solvent; X_3_—water in solvent (%, *v*/*v*).

The coefficient plots illustrate the magnitude and direction of influence for each factor for the considered response (Figure 3). For TPC extraction, the highest influence was noticed for the percentage of water in acetone, whereas the solvent was the second most influential factor. In the case of TFC and CTC, the solvent had the highest impact, but only in CTC did the percentage of water in acetone show a real influence.

**Figure 4:**
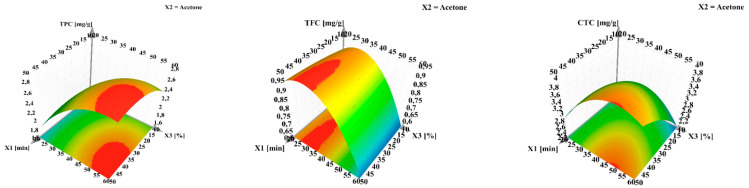
Response surface for predicting the recovery yield for total phenolic content (TPC), total flavonoid content (TFC) and condensed tannin content (CTC) from walnut flower extracts. X_1_—extraction time (min); X_2_—aolvent (cetone); X_3_—water in solvent (%, *v*/*v*). The regions in red represent the domains of working conditions assuring the maximum extraction yield for the evaluated bioactive compounds.

The dependent variables obtained from the screening experimental design can be further investigated by adding higher variation levels to the quantitative factors, thus better depicting the resulting effect. Using contour plots that allowed for curvature modeling, non-linear effects can be visualized for total bioactive content (Figure 4).

#### 3.2.2. The Influence of Experimental Conditions on Antioxidant Activity

**Figure 5:**
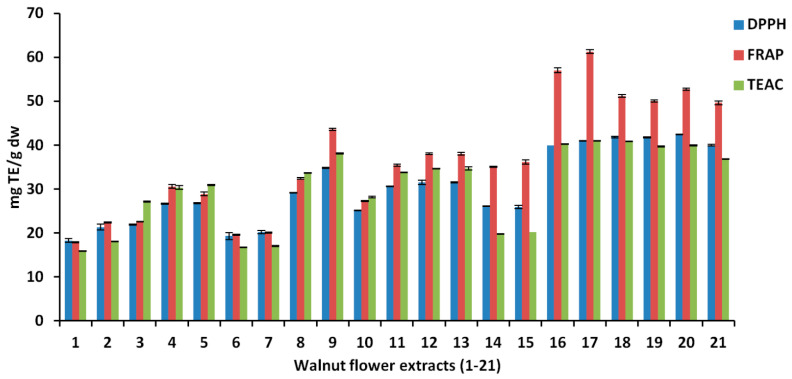
The antioxidant activity evaluated by DPPH, FRAP and TEAC assays for walnut flower extracts.

The results for the antioxidant activity using DPPH, FRAP and TEAC assays for the 21 WMF extracts are depicted in Figure 5.

As in the case of total polyphenol content, the independent variables revealed an important impact on the responses. The values for DPPH, FRAP and TEAC ranged between 40.99–61.34 mgTE/g for acetone, 34.89–43.59 mgTE/g for ethanol and 26.81–30.94 mgTE/g for methanol (Table 2). Again, the highest outcomes were noted for acetone. The best results for DPPH were obtained with 30 min extraction time and 40% water in acetone, revealing a positive relationship with total phenolic content. However, the other two antioxidant assays, FRAP and TEAC, showed the best values at longer extraction times and a higher percentage of water in acetone, at 50 min and 60%, respectively.

**Figure 6:**
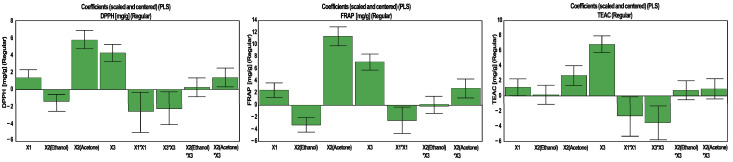
Influence of working conditions on the DPPH, FRAP and TEAC assays of walnut flower extracts, presented as scaled and centered coefficient plots. X_1_—extraction time (min); X_2_—solvent; X_3_—water in solvent (%, *v*/*v*).

The magnitude and direction of influence for each factor are illustrated in Figure 6. Once more, it can be noted that the variables with the highest impact vary between the responses. For DPPH and FRAP, the highest influence was detected for the solvent (acetone) followed by the percentage of water in acetone, whereas the order for the most influential factors is reversed for TEAC.

**Figure 7:**
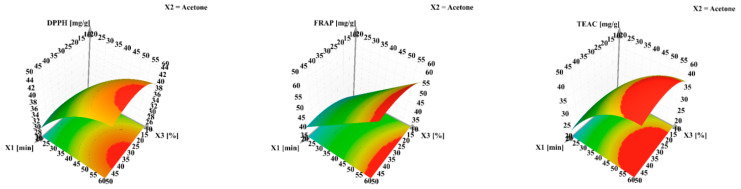
Response surface for predicting DPPH, FRAP and TEAC antioxidant activity in walnut flower extracts. X_1_—extraction time (min); X_2_—solvent (Acetone); X_3_—water in solvent (%, *v*/*v*). The regions in red represent the domains of working conditions assuring the maximum extraction yield for the evaluated bioactive compounds.

The non-linear effects of the examined factors on the antioxidant activities can be very clearly observed in the curvature modeling (Figure 7).

After the investigation of all the evaluated responses and how each factor influenced the extraction yield for the evaluated bioactive compounds and the antioxidant activity, the optimal extraction conditions for each evaluated response were generated using Modde software. Acetone was the solvent with the highest results. The best working conditions with the maximum extraction power for the bioactive compounds and for the DPPH assay are a 30-min extraction time and 40% water in acetone, whereas for FRAP and TEAC analyses, the optimum extraction time and solvent mixture was 50 min and 60% water in acetone, respectively (Table 5).

### 3.3. Phenolic Content and Antioxidant Activity

As previously mentioned, the content of phytochemicals in different parts of a vegetable matrix varies greatly. The synthesis of phytochemicals can be regulated by genetics, including cultivar, and environmental factors such as soil type, sun exposure, rainfall, organic or conventional farming and harvest time [41]. Particularly, environmental stress (i.e., light exposure, drought, pest infestation) can significantly affect the synthesis of polyphenols [42]. Furthermore, the phenolic content of plant extracts is subject to extraction methods, experimental conditions and different ways of presenting the outcomes (for extract, fresh matrix, or dried material). Table 6 summarizes the scientific data found in the literature for the WMF matrix. However, the outcomes have been reported in different units, thus the exact antioxidant activity value cannot be determined from the summarized values.

Zhang et al. [43] analyzed WFs in parallel with the diaphragma juglandis fructus (septum) and walnut pellicle, using three extraction methods—heat reflux extraction (RE) in 70% methanol, UE in 70% methanol and enzymatic extraction (EE) in 70% methanol at pH 5.5, with cellulase. The mean value obtained for TPC in this study was of 1350.77 ± 44.58 mg GAE/g WF. As can be seen, the value is over-dimensioned (1.35 g GAE/g WF) and it cannot be taken into account. The TPC determined in our optimized extract (2.86 mg GAE/g) is close to the values obtained by Rosa et al. for *Juglans regia* from the Modena region, in Italy [40].

Other parts of the walnut tree usually contain higher quantities of TPC. Carvalho et al. found mean value contents of 116.22 mg GAE/g of kernel extract, 94.39 mg GAE/g of leaf extract and 50.18 mg GAE/g of green husk extract, which represented 21.43 mg GAE/g of kernels after accounting for extraction yields [44]. In other studies, TPC ranged from 15.58 to 16.25 mg GAE/g [45,46], and from 4.98 to 21.49 mg GAE/g of kernels, depending on the cultivar and vegetation period [47]. In walnut septum, hull and shell, the TPC mean values were 67.03 ± 9.76 mg GAE/g, 24.68 ± 0.43 mg GAE/g and 18.04 ± 0.42 mg GAE/g, respectively [8,48].

The content of total flavonoid compounds obtained in our study, 0.98 mg QE/g or 98 mg/100 g dried WMF, conforms to other reported data (Table 6). The maximum TFC content (21.5 ± 0.4 mg rutin equivalents/g WMF) was determined in the flowering stage when the inflorescence is already yellow, in contrast to the TPC, which reached the highest content in the early flowering stage [15].

Regarding the flavonoid content in different parts of the walnut tree, it ranged from 5.52 to 28.48 mg QE/g in walnut leaf extracts [49], whereas the highest TFC values determined in walnut septum and walnut skin were 9.76 mg QE/g and 0.52 mg QE/g, respectively [8,50].

Condensed tannins or proanthocyanidins are oligomers and polymers of flavonoids, specifically flavan-3-ols. They are extensively metabolized by gut microorganisms to valerolactone intermediates and hydroxybenzoic acids [51]. Proanthocyanidins are known to scavenge free radicals and inhibit lipid peroxidation and lipoxygenases in vitro, thus having important antioxidant potential [52]. In our experiment, the CTC was 4.13 mg CE/g, whereas the only other study we could find in the literature to quantify proanthocyanidins in walnut flowers showed a value of 38.31 mg CE/g [43]. Knowing that the use of catechin as a standard in plant matrices with a high content of tannins may over- or underestimate their concentration [53], these numbers should be viewed with some cautiousness.

Antioxidant activity has been associated with phenolic content in several studies, with the concentration of polyphenols and flavonoids determining the pharmacological effects of WMF extracts [15]. This trend was revealed in our study, with a good correlation being established between the total polyphenol quantities and the antioxidant activity (Table 2). The highest concentration of trolox equivalent antioxidant activity by DPPH method was seen in sample 20 (30 min extraction time and 40% water in acetone), whereas for the FRAP and TEAC assays the best conditions were 50 min extraction time and 60% water in acetone. On the same matrix, another experiment revealed good results for DPPH and FRAP in ethanolic extracts, 50 min extraction time and 50% water in the solvent [15]. It was suggested in other studies that the number of hydroxyls in the phenol molecule structure was an essential factor in determining the antioxidant capacity of the phenolic compounds [54].

The DPPH radical scavenging capacity of the walnut flower extracts showed a declining trend with the opening of the flower, in relation to the decrease in TPC [15]. The same correlation was exposed in walnut kernels, with the antioxidant potential being greater in the summer months when the phenol content was at the highest level [55]. Compared to other edible flowers (Queen-of-tropic flowers, West Indian jasmine and pagoda trees), the DPPH radical capture activity of WMF was higher, at 84.33% versus 31.39%, 62.52% and 69.65%, respectively [56].

Regarding the ability to reduce ferric ion, previous studies have shown that WMF extracts have superior antioxidant activity compared to other vegetables (spinach, tomato) and fruits (grapes, apple) [15,57]. Mollica et al. performed several assays to determine the antioxidant properties in hazelnut kernels. Employing the ultrasonication extraction method, the best antioxidant values for the hazelnut extracts using DPPH, FRAP and TEAC assays were 22.48 mgTE/g, 24.67 mgTE/g and 48.38 mgTE/g, respectively [58].

### 3.4. Individual Bioactive Compounds

The phenolic compounds and tocopherols identified and quantified in the walnut flower extracts are presented in Table 7.

Analyzing the WMF extracts, a total of 10 polyphenols were quantified, with six polyphenols (p-coumaric acid, ferulic acid, hyperoside, isoquercitrin, quercitrin, quercetin) quantified using the validated LC-MS method, and four polyphenols (catechin, syringic acid, gallic acid and protocatechuic acid) through the LC-MS/MS method. The best solvent in the study was acetone, followed by ethanol and methanol. The highest extraction yield for all the identified polyphenols was obtained at 30 min extraction time, with 40% water in acetone. Because no quantification data were found in the literature regarding individual polyphenols for this matrix, some values for other related matrices were cited.

Catechin, an antioxidant flavonoid, was found in small amounts in WMFs compared to walnut septum, at 15.1 vs. 468.6 µg/g. The same was true for hydroxybenzoic acids: the amounts for syringic acid, gallic acid and protocatechuic acid were 0.9, 8.2 and 1.6 µg/g, respectively. In hazelnut skin extracts, Montella et al. obtained quantities of 62.1 and 21.1 µg/g of for gallic acid and protocatechuic acid, respectively [59]. However, Jakopic et al. found much smaller amounts of gallic and protocatechuic acids in hazelnut kernels—0.52 and 2.92 µg/g, respectively [60].

The amounts of two cinnamic acid derivatives, p-coumaric and ferulic acids, were 440.8 and 49.1 µg/g, respectively. These acids could not be quantified in the walnut septum, but Shahidi et al. obtained for these two acids values of 1662 and 327 µg/g, respectively, in green leafy cover extracts of hazelnuts [61].

Quercetin and its glycosides (hyperoside, isoquercitrin, quercitrin) contain polyphenolic chemical substructures that act as antioxidants by scavenging free radicals that are responsible for oxidative chain reactions [62]. Very important quantities of quercetin (101.9 µg/g), hyperoside (2662.9 µg/g), quercitrin (405.7 µg/g) and isoquercitrin (1293.7 µg/g) were determined in WMF extracts. In the same matrix, Zhang et al. identified 24 polyphenolic compounds (out of 50 analyzed) using an LC-MS/MS method, but they reported lower quantities for quercetin, quercitrin and isoquercitrin, at 63.83, 84.46 and 25.20 μg/g, respectively [43]. Yan et al. qualitatively analyzed the phenol content of an extract obtained from walnut flowers by means of ultra-high-performance liquid chromatography-hybrid quadrupole time-of-flight mass spectrometry (UHPLC-Q-TOF-MS) and identified 36 polyphenolic and flavonoid compounds [4].

The quantification of tocopherols was also performed in our study. The tocopherols are the natural forms of the essential fat-soluble vitamin E, a powerful antioxidant and anti-inflammatory molecule. Based on scientific data, the European Food Safety Authority (EFSA) acknowledged that vitamin E could protect DNA, proteins and lipids from oxidative damage and maintain the normal function of the immune system, heart and blood circulation, as well as bone health [63]. Furthermore, vitamin E, together with vitamins C and K, may exert beneficial effects on glycemic control in diabetes via the reduction of inflammation and oxidative stress [64]. Sample 15, obtained after 50 min extraction time and with 20% water in acetone, showed the best concentrations of α-tocopherol, γ/β-tocopherol and δ-tocopherol, namely 1.6, 3.1 and 24.7 µg/g, respectively (Table 7). At 29.4 µg/g, the total tocopherol quantity was lower in walnut flowers compared to those found in walnut septum, 65.5 µg/g, and walnut kernel, 195.6 µg/g [10]. However, most of the total tocopherol quantity in WMF was provided by δ-tocopherol (84%). This isoform was revealed to be the most active inhibitor of tumor growth, followed by γ-tocopherol and then α-tocopherol, possibly by trapping reactive oxygen and nitrogen species and inducing apoptosis [65]. To the best of our knowledge, this is the first study that has quantified tocopherols in the male flowers of walnuts.

### 3.5. Biological Activities

#### 3.5.1. Enzyme Inhibitory Assays

Enzyme inhibitory theory is regarded as one of the most beneficial therapeutic techniques for managing global health problems. Natural sources have shown enzyme inhibition activity with better bioavailability, less toxicity and are especially preferred for food, medicinal or cosmetic applications [66]. In this research, we examined the inhibitory properties of WMF extract against tyrosinase and α-glucosidase.

Tyrosinase, a copper-containing enzyme, is involved in several human cellular processes, including the transformation of tyrosine to L-DOPA and the synthesis of melanin or neuromelanin. However, overproduction of melanin may result in hypermelanosis and overproduction of neuromelanin, the latter being involved in the pathogenesis of Parkinson’s disease and related neurodegenerative syndromes [67]. As a result, the inhibition of tyrosinase activity may prevent skin hyperpigmentation and alleviate neurodegeneration.

In the current study, the tyrosinase inhibitory activity of the 40% aqueous acetone extract attained after 30 min (N20) was 60.26 ± 0.16 mg KAE/g, whereas for the other extract (N15) obtained with 20% aqueous acetone it was 32.08 ± 0.28 mg KAE/g. These differences between the inhibitory activities could be related to the polyphenolic content, with N20 extract having a higher polyphenolic content (Table 7). Other related matrices—walnut septum and hazelnut involucre—had higher values, at 129.98 ± 3.03 mg KAE/g [8] and 165.17 ± 1.88 mg KAE/g [39], respectively, whereas hazelnut extract revealed a lower outcome at 2.28 ± 0.01 mg KAE/g [58].

Delayed glucose absorption and control of postprandial hyperglycemia, important antidiabetic strategies, can be achieved using amylase or α-glucosidase inhibitors. The amylases hydrolyze dietary starch to oligosaccharides (disaccharides and trisaccharides), which are further converted by α-glucosidase to glucose [10]. The inhibition of these enzymes is part of the management of diabetes treatment. In this experiment, the α-glucosidase inhibition activity was 79.87 ± 0.08 mmol ACAE/g for N20 and 51.06 ± 0.08 mmol ACAE/g for N15. In a related matrix (hazelnuts), the inhibitory activity of this enzyme was 7.57 mmol ACAE/g of hazelnuts, a much smaller inhibition potential compared to WMF [58]. The anti-enzymatic effect of the walnut flower extracts could be associated with the synergistic action of the bioactive compounds found in this vegetable matrix. Thus, further investigation is required to complete the phytochemical profile and explain the mechanisms of action of this by-product.

#### 3.5.2. Biological Activities on Cell Lines

**Figure 8:**
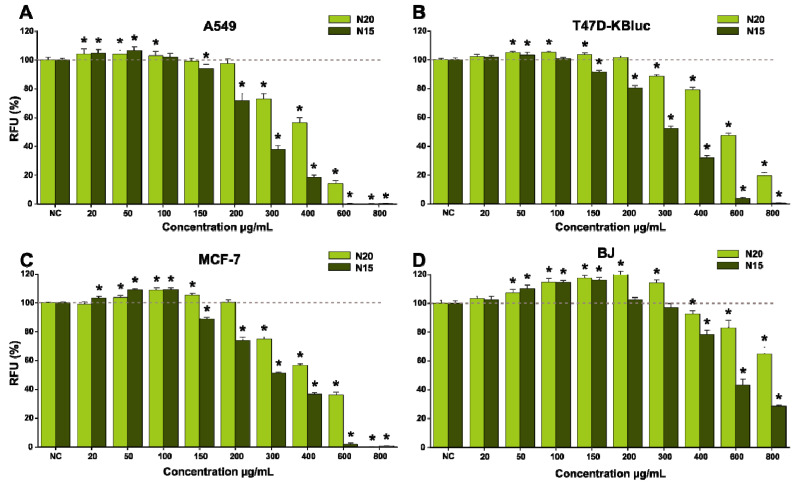
Effects of the *Juglans* sp. extracts (N20, N15) on the cancerous cell lines A549 (**A**), T47D-KBluc (**B**) and MCF-7 (**C**) and on normal human foreskin fibroblasts (BJ) cells’ (**D**) viability after 24 h exposure. The results are presented as relative mean values ± standard deviations, where the negative control (NC) is 100%. Significant differences in comparison with NC (ANOVA + Dunnett’s; *p* < 0.05) are marked with *.

**Figure 9:**
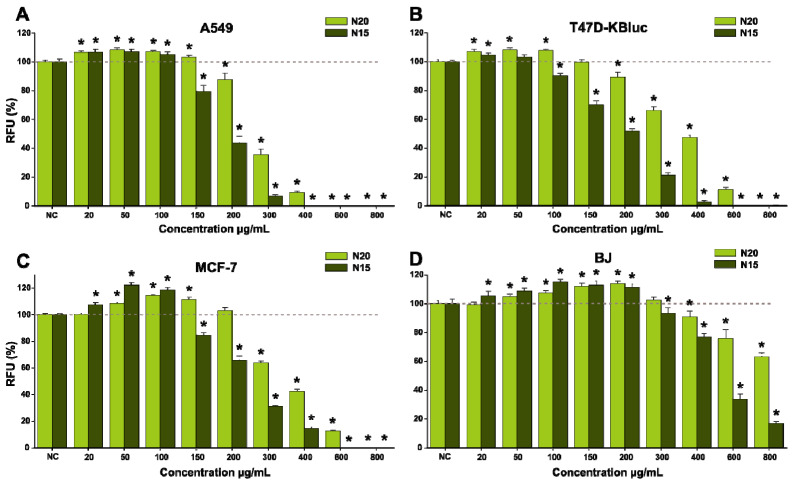
Effects of the *Juglans* sp. extracts (N20, N15) on the cancerous cell lines A549 (**A**), T47D-KBluc (**B**) and MCF-7 (**C**) and on normal BJ cells’ (**D**) viability after 48 h exposure. The results are presented as relative mean values ± standard deviations, where the negative control (NC) is 100%. Significant differences in comparison with NC (ANOVA + Dunnett’s; *p* < 0.05) are marked with *.

The two extracts (N20 and N15) were evaluated for their anticancerous activity on three cancerous cell lines with pulmonary (A549) and mammary gland (T47D-KBluc and MCF-7) origins after being exposed for 24 h and 48 h, respectively. Human foreskin fibroblasts (BJ) were used as a normal cell model to investigate a possible selective cytotoxic effect. Independently of the cell type, exposure to the extracts for 24 h and 48 h resulted in a significant increase in viability at low/intermediary doses, whereas at higher doses a cytotoxic effect was observed (Figure 8 and Figure 9). Both extracts induced the highest increase in viability in the case of the MCF-7 cell line, where exposure to 100 µg/mL N20 and N15 for 24 h resulted in an approximately 10% increase in viability, whereas at 48 h, the increase was approximately 15% for N20 and 20% for N15. To characterize the potency of the extracts, IC_50_ values were calculated based on the dose-effect response. Starting from intermediary doses (>150 µg/mL), a difference in terms of cytotoxicity was observed between the two extracts, with N15 displaying a more pronounced cytotoxic effect (Figure 8 and Figure 9, Table 8). Even though N20 extract was more abundant in polyphenols, flavonoids and condensed tannins, the less water content used in the extraction of N15 increased the extraction yield of lipophilic compounds, as observed in the increased concentrations of tocopherols present in this extract (Table 7). This difference in composition could explain its higher toxicity on the cancerous cells of the latter extract.

A difference in susceptibility was also observed between the cancerous cell lines used, with the A549 cells being the most susceptible to the cytotoxic effects, whereas MCF-7 displayed a higher resilience (Table 8). In line with previous research from our group in which the anticancerous effects of a septum extract were evaluated, and also in line with the literature data, the MCF-7 cell line was the most resilient cell type to the cytotoxic effects of *Juglans* sp. extracts [68]. Regarding the selectivity towards cancerous cell lines, a moderate selectivity was observed, with the human fibroblasts being less affected by the extracts with calculated IC_50_ values for 24 h and 48 h, approximately double those calculated in the case of cancerous cell types (Table 8).

Although other studies have reported the cytotoxic effect of individual compounds (juglone, urolithins, etc.) or various extracts obtained from the green husk, bark, leaf, septum, and kernel of *Juglans* sp., to the best of our knowledge, this is the first study that has evaluated the anticancerous effect of WMF extracts. The results obtained are comparable with the current data on *Juglans* sp., with the reported IC_50_ values ranging from 20 to more than 1500 µg/mL, depending on the extraction method and solvent, as well as on the vegetal parts used for the extraction. The opportunity of natural products derived from *Juglans regia* in the fight against cancer was recently thoroughly reviewed by Catanzaro et al. [41].

**Figure 10:**
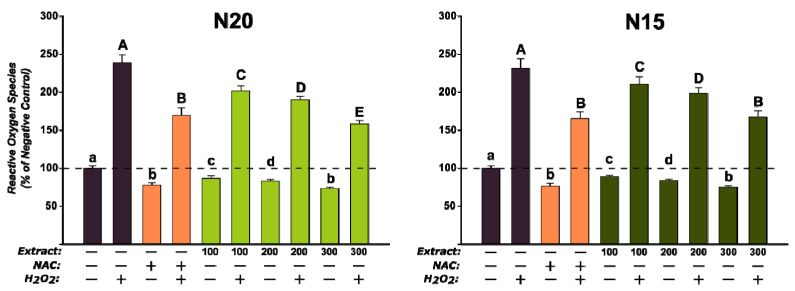
Antioxidant effects of the *Juglans* sp. extracts (N20, N15) using the DCFH-DA assay on normal BJ cells after 24 h exposure. The results are presented as relative mean values ± standard deviations, where the negative control (NC) is 100%. Different letters (a–e refer to comparisons on non-H_2_O_2_ stimulated conditions, whereas A–E refer to comparisona on stimulated conditions) indicate significant differences (ANOVA + Holm–Sidak post hoc test at *p* < 0.05).

The antioxidant effect of the two extracts was assessed on the normal cellular phenotype at 100, 200 and 300 µg/mL concentrations, which were not cytotoxic after an exposure of 24 h (Figure 8). Exposure to the extracts alone decreased the quantity of reactive oxygen species (ROS) in a dose-dependent manner for both extracts, whereas co-exposure to the extracts and 250 µM H_2_O_2_ decreased ROS in a similar matter (Figure 10). The results obtained for both extracts at the highest concentration were similar (N15) or even better (N20) than the response elicited by NAC at a concentration of 10 mM (Figure 10).

In agreement with the current results, Muzzafer et al. reported that a methanolic extract of male flowers from the same species reduced oxidative stress induced by the exposure of human keratinocytes to UV light [69]. Moreover, we previously reported that an extract obtained from walnut septum reduced oxidative stress in several cell lines [10], whereas Muthaiyah et al. reported a protective effect of walnut kernel extract against the oxidative stress induced by exposure to the amyloid-beta peptide in PC12 neuronal cells [70]. The current results also corroborate the data from the literature and the current results obtained from antioxidant assays performed in non-biotic conditions, namely, the FRAP, DPPH and TEAC results (Figure 5) [44,71].

Several compounds present in these extracts, such as tocopherols, quercetin and quercetin glycosides, have been shown to possess antioxidant capacities through the direct neutralization of ROS or by activating the nuclear factor erythroid 2-related factor 2/antioxidant-responsive element (Nrf2/ARE) pathway [72,73,74,75,76,77,78,79,80]. Activation of this pathway leads to the synthesis of cellular antioxidants including glutathione and the increased activity of detoxifying enzymes, thus favoring the ability of cellular functions to cope with the insults induced by ROS [81].

## 4. Conclusions

In the current study, we characterized the phytochemical profile of walnut (*Juglans regia* L.) male flowers and systemically investigated the experimental conditions required for achieving high extraction efficiencies of active phytochemicals from this by-product through accessible and efficient ultrasonic-assisted extraction. To determine the ideal extraction conditions, three parameters (solvent, extraction time and water content in the solvent) were coupled with phytochemical analysis and statistical tools. The most promising extracts were further characterized both qualitatively and quantitatively in terms of individual polyphenolic and tocopherol content. Based on the evaluated polyphenols, quercetin and its quercetin derivatives (hyperoside, quercitrin and isoquercitrin) were the most representative compounds. Even though the total tocopherol concentrations were lower than in other *Juglans regia* parts, most of the total tocopherol quantity in WMF was provided by δ-tocopherol, a highly biologically active isoform. The extracts with the highest polyphenolic content (N20) and tocopherol content (N15), respectively, were further examined for their enzyme inhibitory activities on tyrosinase and α-glucosidase, key enzymes involved in age-related diseases. The results indicated that the extract with the higher polyphenolic content had a higher capacity to inhibit these enzymes, a capacity which is most probably related to polyphenols, as this class of compounds has been shown to inhibit the activities of various enzymes. Moreover, the anticancerous and antioxidant potentials of the two extracts in cellular cultures were evaluated. The tocopherol-rich extract (N15) had higher cytotoxicity on the cancerous cell lines used, whereas a moderate selectivity towards the cancerous phenotype was observed for both extracts. At non-cytotoxic concentrations, both extracts displayed good antioxidant activities in cellular cultures, decreasing the basal and H_2_O_2_-induced oxidative stress.

This study increases the state of knowledge about walnut flowers and, as far as we know, it is the first to describe the tocopherol content and anticancerous potential of this vegetable matrix. The potency of the extracts warrants further scientific investigation in order to understand the bioavailability and the mechanisms of action behind the biological activities of *Juglans regia* male flowers.

## Figures and Tables

**Table 1:** Independent and dependent variables of the experimental design.

Variables	Level		
	−1	0	1
Independent Variables (Factors)			
X_1_—Extraction time (min)	10	30	50
X_2_—Solvent	Methanol	Ethanol	Acetone
X_3_—Water in solvent (%, *v*/*v*)	20	40	60
Dependent variables (responses)			
Y_1_—Total phenolic content (TPC), mg GAE/g ^1^			
Y_2_—Total flavonoid content (TFC), mg QE/g ^2^			
Y_3_—Condensed tannin content (CTC), mg CE/g ^3^			
Y_4_—DPPH antioxidant activity, mg TE/g ^4^			
Y_5_—FRAP antioxidant activity, mg TE/g			
Y_6_—TEAC antioxidant activity, mg TE/g			

^1^ GAE/g (gallic acid equivalents per gram); ^2^ QE/g (quercetin equivalents per gram); ^3^ CE/g (catechin equivalents per gram); ^4^ TE/g (Trolox equivalents per gram).

**Table 2:** Matrix of experimental design and experimental results of walnut flower extracts based on a factorial design.

Exp.	Run	X_1_	X_2_	X_3_	Y_1_	Y_2_	Y_3_	Y_4_	Y_5_	Y_6_
		Extraction Time (Min)	Solvent	Water% (*v*/*v*)	TPC	TFC	CTC	DPPH	FRAP	TEAC
N1	1	10	Methanol	20	0.9049	0.6155	1.1252	18.311	17.861	15.914
N2	18	50	Methanol	20	1.0593	0.6396	1.5492	21.381	22.437	18.127
N3	21	10	Methanol	60	1.3529	0.3574	1.7938	21.885	22.546	27.165
N4	6	50	Methanol	60	1.8835	0.3923	2.2773	26.679	30.615	30.377
N5	10	30	Methanol	40	1.7495	0.5055	1.5885	26.808	28.941	30.949
N6	17	10	Ethanol	20	1.1322	0.7088	0.7704	19.344	19.643	16.771
N7	12	50	Ethanol	20	1.1301	0.7436	0.9503	20.211	20.125	17.080
N8	9	10	Ethanol	60	1.9479	0.5502	1.0403	29.218	32.446	33.685
N9	5	50	Ethanol	60	2.4204	0.5639	3.1770	34.897	43.597	38.158
N10	14	10	Ethanol	40	1.6673	0.8688	1.8837	25.205	27.303	28.164
N11	20	50	Ethanol	40	2.2891	0.9115	1.9962	30.678	35.420	33.828
N12	16	30	Ethanol	20	1.1794	0.7828	0.8378	31.576	38.070	34.708
N13	8	30	Ethanol	60	2.2677	0.5500	1.8837	31.576	38.070	34.708
N14	3	10	Acetone	20	1.5474	0.9248	2.5023	26.169	35.118	19.769
N15	13	50	Acetone	20	1.7240	0.8917	2.8115	25.980	36.184	20.245
N16	4	10	Acetone	60	2.6333	0.5194	1.6026	39.924	57.074	40.276
N17	15	50	Acetone	60	2.6908	0.6025	3.9642	41.013	61.349	40.990
N18	11	30	Acetone	40	2.6505	0.9259	4.0205	41.870	51.221	40.871
N19	19	30	Acetone	40	2.6419	0.9354	4.1329	41.798	50.077	39.729
N20	7	30	Acetone	40	2.6505	0.9276	3.5542	42.456	52.798	40.015
N21	2	30	Acetone	40	2.8591	0.9865	3.8767	40.022	49.667	36.872

Y_1_: TPC—total phenolic content (mg GAE/g); Y_2_: TFC—total flavonoid content (mg QE/g); Y_3_*:* CTC—condensed tannin content (mg CE/g); Y_4_: DPPH—DPPH antioxidant activity (mg TE/g); Y_5_: FRAP—ferric reducing antioxidant power (mg TE/g); Y_6_: TEAC—trolox equivalent antioxidant capacity (mg TE/g).

**Table 3:** Optimization of extraction parameters.

Quantifiable Responses	Source	DF	SS	MS	*F*-Value	*p*
Total phenolic content (Y_1_) (R^2^ = 0.95, Q^2^ = 0.88)	Regression	7	7.85	1.12	38.05	0.01
	Lack of fit	10	0.35	0.035	3.12	0.189
	Pure error	3	0.03	0.01		
Total flavonoid content (Y_2_) (R^2^ = 0.93, Q^2^ = 0.71)	Regression	8	0.72	0.09	21.20	0.01
	Lack of fit	9	0.05	0.01	6.54	0.075
	Pure error	3	0.01	0.00		
Condensed tannin content (Y_3_) (R^2^ = 0.88, Q^2^ = 0.56)	Regression	9	22.68	2.52	9.21	0.01
	Lack of fit	8	2.82	0.35	5.60	0.092
	Pure error	3	0.19	0.06		
DPPH antioxidant activity (Y_4_) (R^2^ = 0.97, Q^2^ = 0.85)	Regression	8	1302.18	162.77	44.14	0.01
	Lack of fit	8	37.24	4.65	4.21	0.132
	Pure error	3	3.31	1.11		
FRAP antioxidant activity (Y_5_) (R^2^ = 0.98, Q^2^ = 0.83)	Regression	7	3342.93	477.56	72.98	0.01
	Lack of fit	9	72.617	8.06	4.10	0.136
	Pure error	3	5.89	1.96		
TEAC antioxidant activity (Y_6_) (R^2^ = 0.96, Q^2^ = 0.89)	Regression	8	1541.38	192.67	38.41	0.01
	Lack of fit	8	46.13	5.76	1.91	0.321
	Pure error	3	9.03	3.01		

DF—degrees of freedom; *F*-value—Fischer’s ratio; MS—mean square; *p*—probability; Q^2^—goodness of prediction; R^2^—coefficient of determination; SS—sum of squares.

**Table 4:** Regression equation coefficients for total bioactive compounds and antioxidant activity in walnut flower extracts.

Effect	Responses					
	Y_1_	Y_2_	Y_3_	Y_4_	Y_5_	Y_6_
	TPC	TFC	CTC	DPPH	FRAP	TEAC
Constant	2.281	0.817	2.808	34.763	39.001	36.124
Extraction time	0.126	0.013	0.417	1.327	2.414	1.141
X2(Ethanol)	−0.009	0.038	−0.392	−1.390	−3.254	0.141
X2(Acetone)	0.329	0.094	0.681	5.781	11.379	2.679
% water in solvent	0.411	−0.116	0.339	4.290	7.144	6.741
X1 × X1	−0.134	−0.005	−0.151	−2.537	−2.473	−2.707
X3 × X3	−0.242	−0.107	-0.431	−2.195	-	−3.586
X1 × X3	0.045	-	0.276	-	-	-
X2(Ethanol) × X3	-	0.012	0.160	0.276	0.0556	0.725
X2(Acetone) × X3	-	−0.013	−0.165	1.390	2.787	0.924

X_1_—extraction time (min); X_2_—solvent; X_3_—water in solvent (%, *v*/*v*); Y_1_: TPC—total phenolic content (mg GAE/g); Y_2_: TFC—total flavonoid content (mg QE/g); Y_3_: CTC—condensed tannin content (mg CE/g); Y_4_: DPPH—DPPH antioxidant activity (mg TE/g); Y_5_: FRAP—FRAP antioxidant activity (mg TE/g); Y_6_: TEAC—TEAC antioxidant activity (mg TE/g). For data in bold, *p*-values were < 0.05, therefore statistically significant.

**Table 5:** Optimum experimental conditions for improved recovery of bioactive compounds with increased antioxidant activity from walnut flower extracts.

Parameters	TPC	TFC	CTC	DPPH	FRAP	TEAC
Extraction time (min)	30	30	30	30	50	50
Solvent	Acetone	Acetone	Acetone	Acetone	Acetone	Acetone
Water in solvent (%)	40	40	40	40	60	60
Predicted	2.92	0.96	4.03	43.92	64.45	42.73
Determined	2.86	0.98	4.13	42.46	61.35	40.99
Bias (%)	2.05	2.08	2.48	3.32	4.81	4.07

TPC—total phenolic content (mg GAE/g); TFC—total flavonoid content (mg QE/g); CTC—condensed tannin content (mg CE/g); DPPH—antioxidant activity (mg TE/g); FRAP—antioxidant activity (mg TE/g); TEAC—antioxidant activity (mg TE/g).

**Table 6:** Phenolic content and antioxidant activity for walnut flower extracts (WMFs) and type of extraction method.

Area	Extraction Conditions	TPC (GAE)	TFC	CTC	DPPH	FRAP	TEAC	Ref
Iran	Percolation in methanol (24 h)	71.7 ± 3.2 mg/g extract	61.7 ± 2.7 mg QE/g extract	-	IC_50_ = 674 ± 27.6 μg/mL	-	-	[17,18]
China	US in methanol (40%, *v*/*v*), 50 min at 50 °C for TPC and AA; ethanol (30%, *v*/*v*), 1 h at 70 °C in water bath for TFC	24.3 ± 0.5 mg/g dw WMF (in EFS)	21.5 ± 0.4 mg RE/g WMF (in FS)	-	84.3% ± 0.1% inhibition (in EFS)	2.1 ± 0.1/100 g dw WMF (in EFS)	-	[15]
China	US in methanol (40%, *v*/*v*), 50 min at 50 °C for TPC and AA; ethanol (30%, *v*/*v*), 1 h at 70 °C in water bath for TFC	3.6 mg/100 g fresh WMF; 2.1 mg/100 g dried WMF	3.2 mg RE/100 g fresh WMF; 1.8 mg RE/100 g dried WMF	-	86% for fresh WMF; 78%–79% for dried WMF	3.8/100 g fresh WMF; 2.1–2.2/100 g dried WMF	-	[16]
China	UE in 70% methanol	1350.8 ± 44.6 mg/g	385.0±16.5 mg CE/g	38.3 ± 1.2 mg CE/g	IC_50_ = 57.3 ± 1.4 μg/mL	IC_50_ = 56.3 ± 1.5 μg/mL	IC_50_ = 42.4 ± 1.2 μg/mL	[43]
India	95% methanol (1:6, w/v) for 48 h at 20 °C	129.8 ± 3.1 mg/g dried material	144.6 ± 2.4 QE mg/g dried material	-	IC_50_ = 66.8 ± 2.1 μg/mL	46.6 ± 4.8 g extract	IC_50_ = 53.9 ± 6.5 μg/mL	[20]
Italy	MAE, DoE 50% ethanol in water (*v*/*v*), for 30 min at 60 °C	3.2 ± 0.5 mg/g fresh WF (one cycle); 5.9 ± 0.2 mg/g fresh WF (3 cycles)	-	-	-	-	-	[40]

AA—antioxidant activity; DoE, design of experiments.

**Table 7:** Phenolic compounds and tocopherols identified in the walnut flower extracts.

		Exp.					
		N2	N4	N7	N9	N15	N20
X_1_	Extraction time (min)	50	50	50	50	50	30
X_2_	Solvent *	M	M	E	E	A	A
X_3_	Water in solvent (%, *v*/*v*)	20	60	20	60	20	40
Y_1_	Catechin	9.8	5.3	8.5	7.4	12.6	15.1
Y_2_	Syringic acid	ND	ND	ND	0.4	0.4	0.9
Y_3_	Gallic acid	4.4	5.9	ND	6.3	5.2	8.2
Y_4_	Protocatechuic acid	1.4	1.4	0.9	1.6	1.1	1.6
Y_5_	p-Coumaric acid	90.5	108.5	ND	122.9	272.8	440.8
Y_6_	Ferulic acid	ND	12.6	ND	ND	32.9	49.1
Y_7_	Hyperoside	332.6	575.2	544.1	556.5	1487.4	2662.9
Y_8_	Isoquercitrin	94.4	348.7	91.3	148.3	217.7	405.7
Y_9_	Quercitrin	196.2	314.1	284.1	289.7	714.1	1293.7
Y_10_	Quercetin	ND	7.8	11.6	12.7	18.8	101.9
Y_11_	α-Tocopherol	0.6	0.5	0.9	ND	1.6	ND
Y_12_	γ/β-Tocopherol	0.3	ND	1.8	ND	3.1	ND
Y_13_	δ-Tocopherol	4.3	0.6	14.4	ND	24.7	0.2

* Solvent: M—methanol; E—ethanol; A—acetone; ND—not determined. All responses are expressed as µg of bioactive compounds per gram (µg/g).

**Table 8:** Calculated IC_50_ (µg extract/mL) values after exposure of A549, T47D-kbluc, MCF-7 and human normal foreskin fibroblasts (BJ) to the two extracts for 24 h and 48 h.

Samples	IC_50_ (µg/mL)							
	24 h				48 h			
	A549	T47D	MCF-7	BJ	A549	T47D	MCF-7	BJ
**N20**	440.4	680.4	574.2	>800	266.6	399.3	349.7	>800
**N15**	260.8	322.1	319.7	466	187.1	199.9	225.2	469

## Data Availability

Data is contained within the article.

## Abbreviations

AA—antioxidant activity; ABTS—2,2′-azinobis-(3-ethylbenzothiazoline-6-sulfonate); ACAE—acarbose equivalents; ANOVA—analysis of variance; CE—catechin equivalents; CTC—condensed tannin content; DCFH-DA—2′,7′-dichlorodihydrofluorescein diacetate; DMSO—dimethyl sulfoxide; DoE—design of experiments; DPPH—2,2-diphenyl-1-(2,4,6-trinitro-phenyl) hydrazine; DTNB—5,5-dithio-bis(2-nitrobenzoic) acid; dw—dry weight; EDTA—ethylenediaminetetraacetic acid; FC—Folin–Ciocalteu; FRAP—ferric reducing antioxidant power; GAE—gallic acid equivalents; HBSS—Hanks’ balanced salt solution; HPLC—high-performance liquid chromatography; KAE—kojic acid equivalents; LC-MS—liquid chromatography–mass spectrometry; LC-MS/MS—liquid chromatography coupled with tandem mass spectrometry; L-DOPA—3,4-Dihydroxy-L-phenylalanine; MT—mushroom tyrosinase; NAC—N-Acetyl Cysteine; NC—negative control; PBS—phosphate-buffered saline; PNPG—p-nitrophenyl-β-d-glucuronide; OS—oxidative stress; QE—quercetin equivalents; PB—phosphate buffer; PLS—partial least squares; ROS—reactive oxygen species; RT—room temperature; SD—standard deviation; Trolox—6-hydroxy-2,5,7,8-tetramethylchromane-2-carboxylic acid; TE—Trolox equivalents; TEAC—Trolox equivalent antioxidant capacity; TFC—total flavonoid content; TPC—total phenolic content; TPTZ—2,4,6-Tri(2-pyridyl)-s-triazine; US—ultrasonication; WMFs—walnut male flowers.
